# *Bacillus subtilis* Protects Public Goods by Extending Kin Discrimination to Closely Related Species

**DOI:** 10.1128/mBio.00723-17

**Published:** 2017-07-05

**Authors:** Nicholas A. Lyons, Roberto Kolter

**Affiliations:** Department of Microbiology and Immunobiology, Harvard Medical School, Boston, Massachusetts, USA; University of Washington

**Keywords:** antagonism, cell-cell interaction, evolution, microbial ecology

## Abstract

Kin discrimination systems are found in numerous communal contexts like multicellularity and are theorized to prevent exploitation of cooperative behaviors. The kin discrimination system in *Bacillus subtilis* differs from most other such systems because it excludes nonkin cells rather than including kin cells. Because nonkin are the target of the system, *B. subtilis* can potentially distinguish degrees of nonkin relatedness, not just kin versus nonkin. We examined this by testing a large strain collection of diverse *Bacillus* species against *B. subtilis* in different multicellular contexts. The effects of kin discrimination extend to nearby species, as the other *subtilis* clade species were treated with the same antagonism as nonkin. Species in the less-related *pumilus* clade started to display varied phenotypes but were mostly still discriminated against, while *cereus* clade members and beyond were no longer subject to kin discrimination. Seeking a reason why other species are perceived as antagonistic nonkin, we tested the ability of *B. subtilis* to steal communally produced surfactant from these species. We found that the species treated as nonkin were the only ones that made a surfactant that *B. subtilis* could utilize and that nonkin antagonism prevented such stealing when the two strains were mixed. The nonkin exclusion kin discrimination method thus allows effective protection of the cooperative behaviors prevalent in multicellularity while still permitting interactions with more distant species that are not a threat.

## IMPORTANCE

Multicellular systems like bacterial biofilms and swarms rely on cooperative behaviors that could be undermined by exploitative invaders. Discriminating kin from nonkin is one way to help guard against such exploitation but has thus far been examined only intraspecifically, so the phylogenetic range of this important trait is unknown. We tested whether *Bacillus subtilis* treats other species as nonkin by testing a single strain against a diverse collection of *Bacillus* isolates. We found that the species in the same clade were treated as nonkin, which then lessened in more distant relatives. Further experiments showed that these nonkin species produced a cooperative good that could be stolen by *B. subtilis* and that treating each other as nonkin largely prevented this exploitation. These results impact our understanding of interspecies interactions, as bacterial populations can interact only after they have diverged enough to no longer be a threat to their cooperative existences.

## INTRODUCTION

Multicellular organisms engage in a number of cooperative behaviors that make this lifestyle advantageous to groups despite being potentially disadvantageous to individual cells. In bacteria, whose biofilms and swarms exhibit many of the hallmarks of multicellularity (1), these cooperative traits often take the form of secreted molecules such as the extracellular matrix, digestive enzymes, and environment manipulators like surfactants. Production costs are often paid by only a subset of cells, while the whole community enjoys the benefits. Over evolutionary time, this imbalance needs to be protected from “cheating” invaders that utilize the resources but do not contribute to them (2). One such protection mechanism is kin discrimination (3), in which only closely related cells are cooperated with, increasing the likelihood that the recipients will reciprocate. Kin discrimination is a widespread phenomenon in many walks of life (4–6) and has been shown to prevent exploitation by cheater cells in the microbial eukaryote *Dictyostelium discoideum* (7, 8).

We recently found that the soil bacterium *Bacillus subtilis* exhibits kin discrimination behavior in the context of swarming (9, 10). Swarming is a form of cooperative migration across a semisolid surface (such as a plate with 0.7% agar) in which cells secrete surfactants and hyperflagellate to more quickly access new territory (11). Systematic pairwise tests of swarms of wild *B. subtilis* isolates revealed that interstrain antagonism strongly correlated with genetic relatedness, indicating differential treatment of kin. Additionally, the cutoff between kin and nonkin in this organism is very narrow: strain pairs with <99.5% housekeeping gene identity never recognized each other as kin (10). The high relatedness requirement is due to the combinatorial nature of this particular kin discrimination system, which uses many antimicrobial genes and immunities that vary considerably among strains of *B. subtilis* (9). Groups of cells that do not share a recent ancestry will most likely not possess the ability to produce the exact same combination of these molecules and will be killed instead of cooperated with.

The kin discrimination system in *B. subtilis* differs from other systems in that it is determined by nonkin exclusion rather than kin association, which is how many previously described microbial kin discrimination systems work. In these systems, preferential association with kin is typically mediated by allele-specific interactions between transmembrane receptors. This has been documented in social amebae (12), budding yeast (13), the bacterium *Myxococcus xanthus* (14), and colonial marine invertebrates (15) and even has analogy to neuron self-avoidance in brain development (16). On the other hand, *B. subtilis* (and possibly *Proteus mirabilis* [17–19] as well as a secondary mechanism in *M. xanthus* [20, 21]) instead produces a plethora of diverse antibiotics and toxins to create a barrier that only close relatives can survive (9). Kin are thus identified indirectly by directly targeting and killing nonkin.

In addition to the way that they identify close relatives, the two systems—kin association and nonkin exclusion—differ in their treatment of distantly related organisms. The kin association system treats all cells that are not kin the same regardless of phylogenetic distance—they do not meaningfully interact because they do not have identical recognition molecules (e.g., transmembrane receptors). Kin discrimination in this system is thus targeted intraspecifically (22, 23). The nonkin exclusion system, however, is explicitly directed toward nonkin and thus could potentially distinguish between close and distant nonkin. One reason to believe that this occurs is that *B. subtilis* can be found in multispecies communities (24, 25), and so at some genetic distance, it must be able to coexist with other species, which can often be beneficial to biofilms (26). We note here that our use of the term “nonkin” throughout this work is reserved for the strains that exhibit antagonism toward each other, while nonantagonistic strains are referred to as “distant species” (or similar derivatives) even though they too are technically “not kin.” This is because “nonkin” is juxtaposed with “kin” and thus makes clear that these are cells that are not to be cooperated with, while “distantly related” is meant to suggest that they are not subject to kin discrimination rules.

We therefore tested where the phylogenetic endpoint of kin discrimination behavior is for *B. subtilis*. We hypothesized that interactions with close species would still be dominated by nonkin antagonism but at some phylogenetic distance would shift to a range of interactions that no longer correlated with relatedness. This is indeed what we found: all the other species in the immediate *subtilis* clade were treated as nonkin, after which the interactions transitioned to a random mixture of hostile and nonhostile behaviors. We also found that the antagonistic nature of interspecies interactions correlated with the utilization of surfactant, a public good necessary for multicellular swarming, indicating that the broadening of nonkin behaviors to nearby species could protect cooperative behaviors or at least coincides with compatible cooperative behaviors in these species.

## RESULTS

### Interspecies interaction assays.

**FIG 1:**
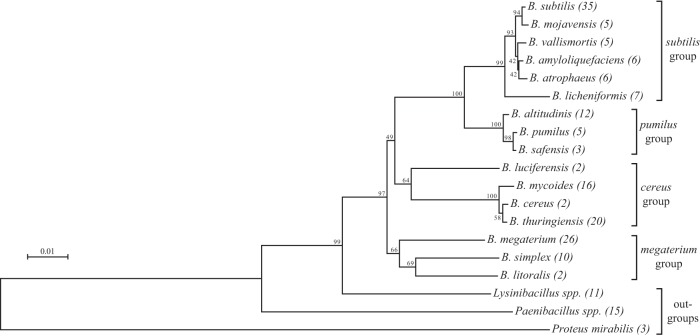
Phylogenetic tree of species used in this study. Minimum-evolution tree based on full-length 16S rRNA gene sequences from the type strain of each species. Unless indicated, all are from the genus *Bacillus*. Three *Lysinibacillus* and five *Paenibacillus* species were used in experiments, but only the sequences from *L. fusiformis* and *P. taichungensis* were included in this tree. In parentheses are the numbers of strains of each species/genus used. Bootstrap values are based on 500 replicates.

In order to determine the phylogenetic breadth of *B. subtilis* kin discrimination, we tested the undomesticated strain *B. subtilis* NCIB 3610 against a diverse panel of strains in various multicellular interaction assays. We tested a collection of 191 wild strains from 19 different species, with a median of six strains per species (Fig. 1). Of these, 35 strains were from stock centers, three were previously isolated by our lab, and 153 were newly isolated for this study (140 of which came from just two ~1-cm^3^ soil samples). Most of these were other closely related *Bacillus* species, but we also included outgroups from two genera (*Lysinibacillus* and *Paenibacillus*) from the same order (*Bacillales*) as well as three strains of *Proteus mirabilis*, a well-studied Gram-negative bacterium that also uses antagonism to discriminate self from nonself in multicellular swarms (17–19). Because kin discrimination behavior has previously been established intraspecifically (10), 35 new *B. subtilis* isolates were included to provide a proven nonkin set to which to compare the interspecies results.

**FIG 2:**
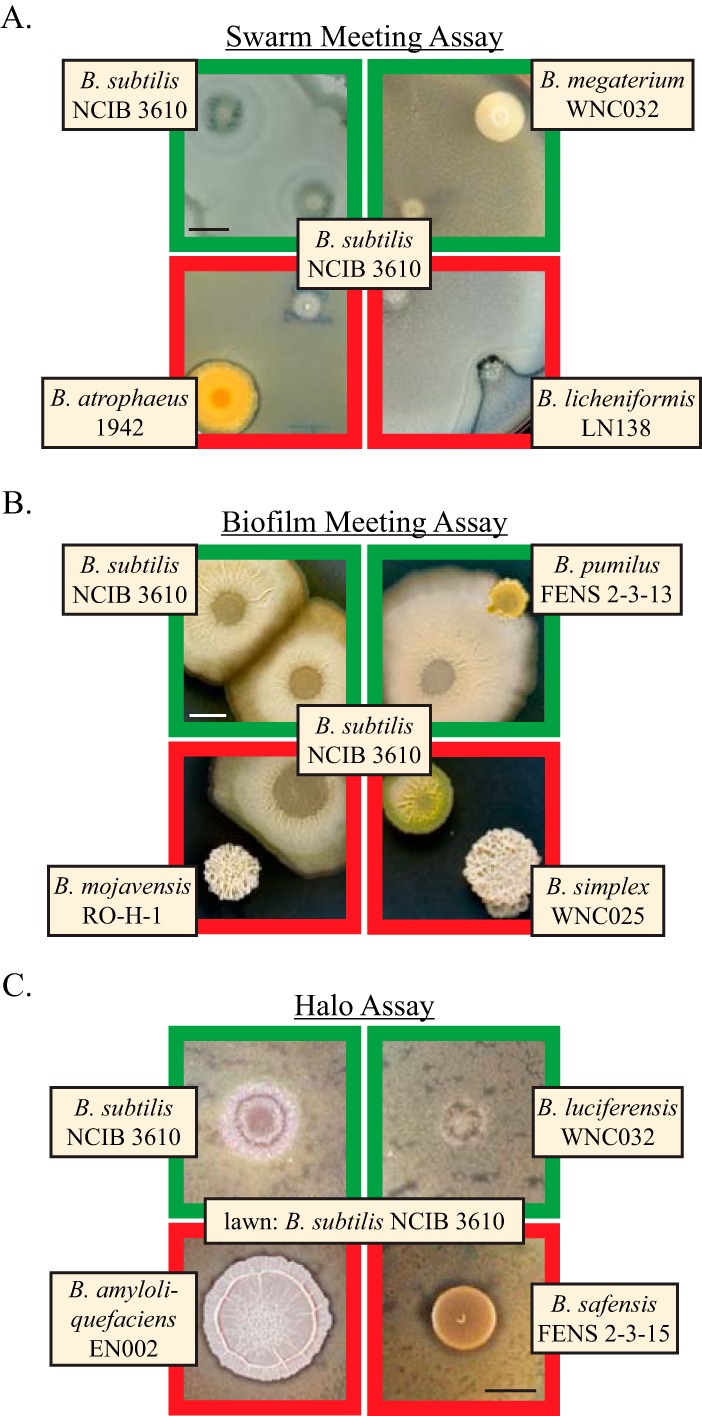
Multicellular interaction assays used to assess kin versus nonkin. (A) Swarm interaction assay. Representative examples of *B. subtilis* NCIB 3610 swarming toward either itself (upper left) or a different species, resulting in either merging of the colonies (top two images, green borders) or formation of a boundary between them (bottom, red borders). Bar, 1 cm. (B) Biofilm meeting assay. Biofilms of *B. subtilis* that encountered or enveloped the indicated species were scored as nonantagonistic (top, green borders), while biofilms that stopped short of the other species or showed signs of impaired growth were counted as antagonistic interactions (bottom, red borders). Bar, 0.5 cm. (C) Halo formation assay. Colonies of the indicated species were spotted on biofilm-inducing medium after top-spreading (but not pregrowing) *B. subtilis* cells and then examined for inhibition of lawn growth (halos) around the colony. Bar, 0.5 cm.

To properly test for kin discrimination behavior, it was necessary to show a correlation between interaction behavior and phylogenetic relatedness (using 16S rRNA gene identity). We therefore employed three different assays (Fig. 2) to evaluate pairwise interactions between our panel of strains and *B. subtilis* NCIB 3610 in different multicellular contexts. A final overall phenotype was then assigned to each strain based on its aggregate behavior in the three assays. Importantly, all 35 of the *B. subtilis* strains displayed obvious antagonism in all three assays, indicating that each assay is a reliable test of nonkin relationships.

First, we performed the same swarming assay that we used to first identify *B. subtilis* kin discrimination (10) that is similar to other bacterial swarming assays used to test recognition (27, 28). Nonkin strains are easily identified by a distinct boundary between their swarm fronts caused by extensive cell death from incompatible antimicrobial complements (9). Kin, conversely, do not kill each other and merge their swarm fronts (visualized using a constitutively expressed yellow fluorescent protein [YFP] in *B. subtilis*). These phenotypes apparently extend to other species as well, as we found both merging and boundary formation behaviors between *B. subtilis* and our panel of strains (Fig. 2A; see full results in Data Set S1 in the supplemental material). Strains from several *Bacillus* species that were not able to swarm required a day of pregrowth to establish a colony before we could properly judge the interaction with *B. subtilis* (e.g., *Bacillus megaterium* and *Bacillus atrophaeus* in Fig. 2A). Pregrown strains still displayed a range of interaction phenotypes, and so their inability to swarm did not affect the results of the assay. Interestingly, while swarms of both *B. subtilis* and *P. mirabilis* exhibit antagonism toward members of their own species (9, 17), they do not do so to each other, as their swarms showed extensive overlap and coexistence in our assay (see Data Set S1). This supports our original hypothesis that nonkin exclusion systems are able to discriminate among close relatives while maintaining coexistence with distant species.

10.1128/mBio.00723-17.3 DATA SET S1  All interaction phenotype data. Results from each assay (swarm, biofilm, halo, and surfactant stealing) of every strain tested, as well as the accession numbers of 16S rRNA gene sequences used to construct trees and calculate percent identities. Download DATA SET S1, XLSX file, 0.1 MB. Copyright © 2017 Lyons and Kolter. 2017 Lyons and Kolter This content is distributed under the terms of the Creative Commons Attribution 4.0 International license.

We next tested each pairwise interaction in two assays that required merely growth on a biofilm-promoting medium and not active swarming ability (29). The first of these assays was predicated on the phenotype of two biofilms meeting on the plate, with a similar readout as the swarming assay. If the colonies contacted each other without obvious growth inhibition, it was judged a permissive (nonantagonistic) interaction; if they did not fully approach one another or if cell death was apparent, antagonism was assumed (Fig. 2B). Phenotypes ranged greatly from complete merging and overlap of both strains, to growth around but not on top of the other colony, to small gaps, to growth inhibition from centimeters away. Differences in growth rates again required some strains to be pregrown for a day or two before addition of *B. subtilis* (e.g., *B. pumilus* and *B. simplex* in Fig. 2B), but all strains were able to form a colony of sufficient size to assess its interaction phenotype. While it is possible that some boundaries between biofilms could be due to nutrient depletion in the medium (each assay took between 2 and 10 days), nearly all cases of antagonism were very apparent from the growth pattern and morphology of both strains (for example, the interaction with *B. mojavensis* RO-H-1 in Fig. 2B).

The third and final multicellular interaction assay was performed on the same MSgg medium as the biofilm meeting assay but was instead based on the effect that a strain had on the growth of a lawn of surrounding *B. subtilis*. Here, antagonistic interactions appeared as a halo of impaired *B. subtilis* growth around the colony, while the lack of a halo indicated coexistence (Fig. 2C). Most colonies without halos also had *B. subtilis* visible inside the colony, as seen by the yellow fluorescent protein (YFP) expressed by *B. subtilis*, suggesting that the absence of a halo is indeed a good indicator of the ability to coexist. Lawn growth was monitored from initial appearance (~15 h) until full density (~40 h) to observe any subtle defects in *B. subtilis* growth, though most halos were observable even when the lawn was overgrown.

### Varied phenotypes among assays.

**FIG 3:**
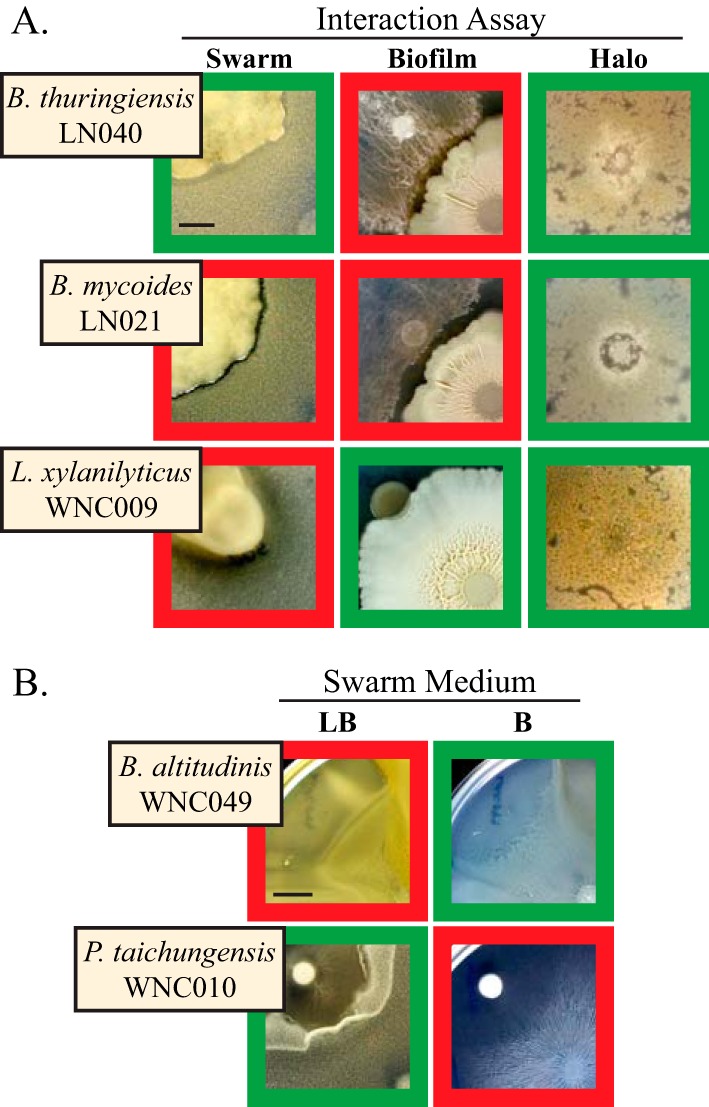
Varied phenotypes between and within different assays. (A) Examples of strains that displayed different phenotypes in the three multicellular interaction assays. Red and green borders indicate interactions judged as antagonistic and not antagonistic, respectively, in the individual assays, leading to a varied score overall. *B. subtilis* is in the lower right corner in the swarm and biofilm images and is the lawn in the halo images. Bar, 0.5 cm. (B) Strains that displayed opposite phenotypes in the swarm assay on different types of medium (LB or B, both with 0.7% agar). This behavior was observed in two strains each of *B. altitudinis* and *Paenibacillus taichungensis* (four strains total). *P. taichungensis* formed a thin swarm on LB that merged with *B. subtilis* (thick white ring) but did not spread out on B medium and formed a wide zone of inhibition. The *B. subtilis* swarm was spotted in the lower right corner in each image. Bar, 1 cm.

Testing each pairwise interaction in multiple different assays allowed us to better determine the overall phenotype of the species as a whole by replicating each result in a different setting. The three assays generally agreed with each other, but they were not always consistent (Fig. 3). For example, many strains with merged swarms produced marked gaps between biofilms (Fig. 3A, top). Strains exhibiting differences between assays were labeled as “varied” phenotype and were unexpectedly common among interspecies interaction types.

These varied differences were found in all combinations, i.e., there were many examples of each assay disagreeing with the other two assays (Fig. 3A). The swarm and halo phenotypes were slightly more correlated with each other (Spearman *r* = 0.6975) than with the biofilm phenotype (swarm *r* = 0.2796, halo *r* = 0.3797), despite the fact that the halo and biofilm assays were done on the same medium. This could be due to many factors: temporal changes in physiology or development (the swarm and halo assays took a single day while biofilm assays lasted up to a week), depletion of nutrients in the medium, or cell density upon meeting (strains first encountered each other at far lower cell numbers in swarms and halos than in thick biofilms). Additionally, the varied phenotypes are representative of the ambiguous literature on whether cooperation or competition dominates interspecies interactions (30–32). Our results suggest that context is an important factor in determining the answer to this question and may even indicate that the answer is often “both.”

Taking the average assay score of each species revealed interesting patterns among the different clades (see Fig. S1A in the supplemental material). Strains in the *pumilus* group tended to have very hostile interactions with *B. subtilis* in swarms and halos but not between biofilms. Conversely, species in the *cereus* clade had antagonistic biofilm interactions on average but coexisted well with *B. subtilis* in the swarm and halo assays. This speaks to the importance of testing interspecies interactions in multiple ways and in multiple settings to more fully capture the various lifestyles of bacteria and their responses to different environments. We even found a few rare strains that had different phenotypes within the same assay (Fig. 3B). In two strains each of *B. altitudinis* and *Paenibacillus taichungensis*, swarms encountering *B. subtilis* reacted oppositely on LB versus B medium. This again underscores the danger in relying on single experiments to accurately judge the nature of an interspecies interaction and reinforced our confidence in the overall assessment of each species’ interaction phenotype.

**FIG 4:**
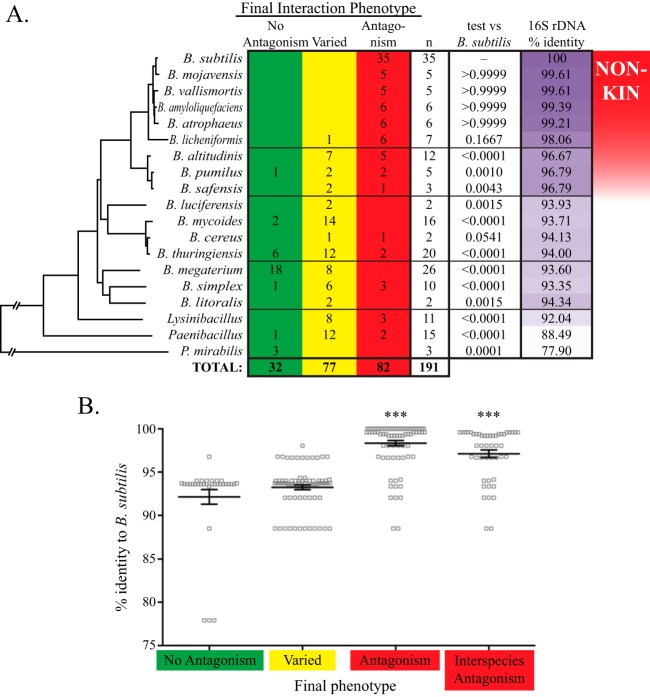
Overall phenotypes from all three interaction assays, arranged by phylogeny. (A) The first three columns indicate the number of strains of each species with the given final phenotype, a sum of the phenotypes from the three assays. The tree is from Fig. 1, except that the *P. mirabilis* branch is abbreviated for brevity; horizontal lines in the table divide clades. Mann-Whitney two-tailed independent tests were performed comparing the set of *B. subtilis* phenotypes (35 antagonistic) to the set from each species; significant *P* values are in bold. The percent identities of 16S rRNA genes to *B. subtilis* are given in the last column, and the red gradient on the far right is the inferred phylogenetic range of kin discrimination behavior. (B) 16S rRNA gene identity to *B. subtilis* of strains in each interaction phenotype group. “Interspecies antagonism” refers to all the non-*subtilis* species that exhibited antagonistic interactions. Bars represent the averages ± standard errors of the means (SEM). Asterisks indicate a significant difference from both the no-antagonism and varied phenotypes (*P* < 0.0001), which are not different from each other (*P* = 0.2516).

10.1128/mBio.00723-17.1 FIG S1  Related to Fig. 4. Average interaction phenotypes of each species. (A) Quantized version of interaction phenotypes, where no antagonism was 0, varied was 0.5, and antagonism was 1. The average scores from each assay with standard errors of the means (SEM) were calculated for each species and for all species and all interspecies (non-*subtilis*) interactions. Final scores were calculated from each strain’s scores in the three assays: strains with three antagonistic results had a final score of 1, three no-antagonism results were given a final score of 0, and strains with differing results in the assays were assigned a final score of 0.5. Horizontal lines divide clades. (B) The same summary of interaction phenotypes as in Fig. 4A, except reported as the number of negative interactions (“neg”) observed in the three assays. This divides the varied category (yellow) into two subcategories depending on whether a strain displayed one or two antagonistic interactions. (C) Graphical view of the average final score of each species from panel A, arranged by increasing relatedness to *B. subtilis* from left to right. Error bars represent the SEMs. Asterisks indicate the species that are significantly different from *B. subtilis* as determined in Fig. 4A. Download FIG S1, EPS file, 2.4 MB. Copyright © 2017 Lyons and Kolter. 2017 Lyons and Kolter This content is distributed under the terms of the Creative Commons Attribution 4.0 International license.

### Correlation of interaction phenotype with phylogeny.

After testing of each strain in all three assays, a final overall phenotype was assigned to each strain: no antagonism, varied, or antagonism (Fig. 4A and S1A; Data Set S1). Final phenotype designations of no antagonism and antagonism required uniform responses toward *B. subtilis* in all three assays, while the varied overall phenotype was assigned whenever there were differences between assays (as described above). The varied phenotype is actually a combination of two subcategories (strains with one antagonistic and two nonantagonistic interactions and vice versa), but we feel that these subcategories are much more similar to each other than to the uniform-response categories. This breakdown of the varied phenotype into its constituent phenotypes is shown in Fig. S1B, however, and shows a similar pattern.

The distribution of the final interaction phenotypes within each species was used to evaluate the overall treatment of that species by *B. subtilis*. All the nearby species in the *subtilis* clade exclusively showed antagonism with *B. subtilis* (Fig. 4A), except for one strain of *B. licheniformis* (the least-related species of the clade that branched away from the other members in all phylogenetic trees constructed) whose biofilms were able to merge with *B. subtilis*. The *B. licheniformis* phenotype distribution was not statistically different from that of *B. subtilis*, however (*P* = 0.1667, Mann-Whitney test). The most closely related species are therefore treated just like intraspecies nonkin, demonstrating carryover of kin discrimination behaviors into interspecies dynamics.

As the species became less related to *B. subtilis*, however, the net phenotype started to shift away from pure antagonism. The closest non-*subtilis* clade, containing *B. altitudinis*, *B. pumilus*, and *B. safensis*, was the first to exhibit varied phenotypes, though many strains were still entirely antagonistic (Fig. 4A), and even the varied strains were heavily weighted toward the two-negative-interaction subcategory (Fig. S1B). There was one strain of *B. pumilus* (FENS 2-3-13 [Fig. 2B]) that never showed signs of hostility, though this strain seems to be an outlier in this clade. All three species displayed a set of phenotypes statistically different from that of *B. subtilis*. However, the distribution of *B. altitudinis* phenotypes is also significantly different from representatives of other clades, *B. mycoides* (*P* = 0.0038), *B. thuringiensis* (*P* = 0.0097), and *B. megaterium* (*P* < 0.0001), as well as the sum total of all strains’ phenotypes outside the *subtilis* and *pumilus* clades (*P* = 0.0020). The *pumilus* group therefore represents a transition between phenotypes: some strains have diverged enough to not be as thoroughly affected by the *B. subtilis* antimicrobial suite, and yet many are still not able to coexist. The idea that the *pumilus* group represents “transition” species fits well with the *Bacillus* phylogeny (Fig. 1), in which the best bootstrap support came from the branch points separating the *subtilis* and *pumilus* clades from the rest of the tree and from each other.

Beyond the *pumilus* clade, most species exhibited a seemingly random mixture of interaction phenotypes that were significantly different from those of *B. subtilis* (Fig. 4A). This included a lot of differences between assays, manifested as a high preponderance of varied phenotype classifications. We interpret this to mean that once a species drops below a certain level of relatedness, it is no longer subject to the purely adverse effects of kin discrimination. What is left is a range of ecological interactions, which can vary widely from cooperative to spiteful but do not necessarily correlate with relatedness. Tellingly, the outgroup genera *Lysinibacillus* and *Paenibacillus* are each dominated by varied phenotypes. *B. megaterium* and *P. mirabilis* seem to be exceptions to the preponderance of varied phenotypes, however, as they were heavily weighted toward no antagonism (although only a few *P. mirabilis* strains were tested).

Overall, the observed phenotypes strongly correlated with phylogenetic relatedness. Plotting the average phenotype score for each species confirmed a distinct decline in antagonism outside the *subtilis* clade (Fig. S1C). Likewise, calculating the average 16S rRNA gene sequence identity to *B. subtilis* revealed a considerable difference between the antagonism category (average identity, 98.35%) and both the no-antagonism (92.14%) and varied (93.24%) categories but no statistical difference between no antagonism and varied (Fig. 4B). If we exclude the 35 *B. subtilis* strains tested, so as to focus the analysis only on interspecies interactions, the average 16S rRNA gene identity of antagonism drops to only 97.13% and statistical comparisons to the other phenotypes remain highly significant (Fig. 4B). Altogether, our data indicate that *B. subtilis* maintains nonkin antagonism toward the other species in its immediate clade, which tapers off through the *pumilus* clade, and by the *cereus* clade, the species are no longer subject to kin discrimination from *B. subtilis*. The correlation of interaction phenotypes with broader phylogeny thus reveals the full phylogenetic range of the nonkin exclusion system of kin discrimination in this bacterium.

### Correlation of kin discrimination with utilization of public goods.

After finding the phylogenetic endpoint of kin discrimination behavior, we looked for factors that might cause that point to be where it is, around the *pumilus* clade. In other words, we wanted to find other traits that correlated with the observed interaction phenotypes that could provide evolutionary reasons for their particular pattern.

Our hypothesis stems from the observation that the *Bacillus* species capable of swarming were those most closely related to *B. subtilis* (Table 1, left). In other words, swarming ability, like nonkin designation, correlates with phylogeny, and so we investigated whether there was a link between them. Swarming is a cooperative behavior that requires the secreted public good surfactin, an amphipathic lipopeptide that reduces friction at the leading edge of the swarm by reducing the water surface tension. Surfactin is produced by enzymes encoded in the *srfAA* operon in *B. subtilis* (33) and can act as a signaling molecule in addition to its physical properties (34). There is also some species specificity among surfactants, as variants of surfactin do not elicit equally robust effects across species (35) and a secreted factor from *Paenibacillus* has little effect in *B. subtilis* or even another *Paenibacillus* species (36). We therefore wondered whether kin discrimination behavior correlates with swarming proficiency, possibly protecting this diffusible common good from exploitation by other swarmers.

**TABLE 1:** Swarming and surfactant-stealing ability of each species

Species	Swarming ability			Usable surfactant^a^		
	No. of strains		% of strains	No. of strains		% of strains
	With	Without		With	Without	
*B. subtilis*	34	1	97	34	1	97
*B. mojavensis*	4	1	80	0	5	0
*B. vallismortis*	3	2	60	2	3	40
*B. amyloliquefaciens*	5	1	83	5	1	83
*B. atrophaeus*	2	4	33	0	6	0
*B. licheniformis*	6	1	86	6	1	86
*B. altitudinis*	11	1	92	7	1	88
*B. pumilus*	5	0	100	4	1	80
*B. safensis*	3	0	100	3	0	100
*B. luciferensis*	0	2	0	0	2	0
*B. mycoides*	0	16	0	0	6	0
*B. cereus*	0	2	0	0	2	0
*B. thuringiensis*	1	19	5	0	8	0
*B. megaterium*	0	26	0	0	6	0
*B. simplex*	0	10	0	0	4	0
*B. litoralis*	0	2	0	0	2	0
*Lysinibacillus*	8	3	73	0	11	0
*Paenibacillus*	8	7	53	0	8	0
*P. mirabilis*	3	0	100	0	3	0
Total	93	98	49	61	71	46

a Elicited spreading from *B. subtilis* NCIB 3610 Δ*srfAA* spotted 3 cm away on the same plate.

Support for this idea comes from the conservation patterns of the lipopeptide synthetases that produce various surfactants (Fig. S2). There are three main families of lipopeptides used by *B. subtilis*: surfactins, iturins, and fengycins (33). Variants within the surfactin family include lichenysin and pumilacidin (first found in *B. licheniformis* and *B. pumilus*, respectively), which differ at a couple of amino acid residues in the lipopeptide but have similar chemical properties (37). The conservation of the three families mirrors our interaction data in that they are well conserved throughout the *subtilis* and *pumilus* clades but not in more distant species (Fig. S2), much like the pattern of nonkin treatment. This is in contrast to the conservation pattern of the extracellular matrix proteins that are found beyond the nonkin range (Fig. S2), at least out to the *cereus* clade (38), which might be because they are not as publicly available as the surfactants (39).

**FIG 5:**
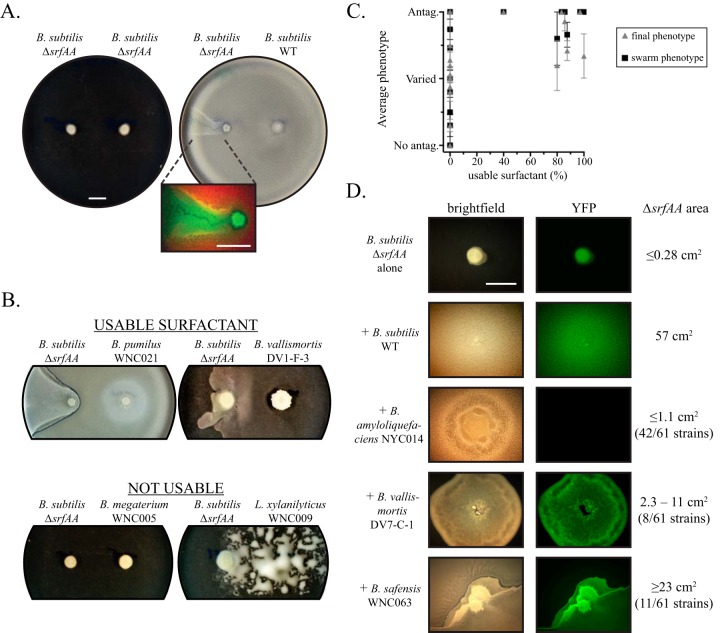
Surfactant-stealing assays. (A) *B. subtilis* NCIB 3610 Δ*srfAA* (constitutively expressing YFP) spotted on swarm-inducing medium 3 cm away from either itself or its wild-type (WT) parent (expressing red fluorescent protein). The enlarged image shows the spatial distribution of the two fluorescent strains (mutant in green, wild type in red; overlap shows up as yellow). (B) Examples of strains that did and did not elicit spreading of *B. subtilis ΔsrfAA*, implying production of a surfactant that the mutant could use. Strain identity was verified by fluorescence as in panel A. (C) Correlation between the percentage of each species that had a usable surfactant and the average phenotype of each species in both the swarm interaction assay (black squares) and the final overall phenotype from all three assays (gray triangles). Values can be found in Table 1 and Fig. S1A in the supplemental material; error bars represent the standard error of the mean for the phenotype score. (D) Micrographs of mixtures of *B. subtilis ΔsrfAA* (expressing YFP) with strains that elicited spreading as determined in panel B. Examples are shown from the three general phenotype categories as defined on the right: zero/little spreading (0-cm^2^ area shown), modest spread (5.7 cm^2^ shown), and good spread but spatial segregation (24 cm^2^ shown). The number of strains that exhibited each phenotype is indicated in parentheses on the right. All bars, 1 cm.

10.1128/mBio.00723-17.2 FIG S2  Related to Fig. 5. Conservation of surfactant-producing enzymes correlates with kin discrimination range. Conservation of the proteins that make secreted lipopeptides and extracellular matrix used by *B. subtilis*, based on BLASTp searches of each species. Conservation assessment is based on the most typical found in that species, but proteins are not always shared among all strains of that species. See Materials and Methods for the proteins used as search criteria for each lipopeptide family and the percent identities used to define the presence/absence of homologs. Similar results were also obtained from BLASTn searches of DNA sequences of the full operons (data not shown). Note that species have very uneven representation in the searched databases (especially *B. luciferensis* and *B. litoralis*). Phylogenetic tree and nonkin range are from Fig. 4. Download FIG S2, EPS file, 1.1 MB. Copyright © 2017 Lyons and Kolter. 2017 Lyons and Kolter This content is distributed under the terms of the Creative Commons Attribution 4.0 International license.

To test our hypothesis of public good protection, we developed a surfactant-stealing assay using an *ΔsrfAA* mutant of *B. subtilis* NCIB 3610 that cannot produce surfactant and cannot swarm on its own (40). We first assessed whether the strains in our collection produced surfactants that could be used by *B. subtilis*. In this assay, *B. subtilis ΔsrfAA* cells were spotted a few centimeters away from another strain on swarm-inducing medium to see if the mutant could use surfactant secreted by the other strain to initiate swarming, as surfactants can diffuse rapidly across large distances. *B. subtilis ΔsrfAA* was marked with constitutively expressed YFP to allow easy visualization of its spread and distinguish it from the other strain. When the mutant was spotted next to another *ΔsrfAA* strain, neither strain spread out at all (Fig. 5A, left). In contrast, when spotted next to its wild-type parent the mutant was able to steal the surfactin and spread out ahead of the advancing wild-type swarm in a comet-like pattern (Fig. 5A, right). This pattern is likely a consequence of the lag time between initiating swarm development and actually spreading out (11). Importantly, however, the fluorescent overlay in Fig. 5A shows that only the mutant strain (false-colored green) is present in the comet tail, indicating that the mutant is indeed swarming on its own and not merely spreading on top of wild-type cells.

Thus, strains that elicited spreading of the *ΔsrfAA* mutant were inferred to produce a surfactant that *B. subtilis* could exploit. Such strains were categorized as “usable surfactant.” The opposite effect—not stimulating mutant spread—was labeled “not usable surfactant,” which is not meant to imply anything about the presence or absence of lipopeptides in the strain but merely the ability of *B. subtilis* to utilize them for swarming.

We submitted the majority of our strain collection (132 of 191 strains, at least four from each species) to this surfactant production assay. Overall, 61 strains (46%) caused *B. subtilis ΔsrfAA* to spread, but all were species from the *subtilis* and *pumilus* clades (Fig. 5B; Table 1, right). Significantly, no strains from the distant outgroups that are excellent swarmers (*Lysinibacillus*, *Paenibacillus*, and *P. mirabilis*) elicited any response from the mutant, indicating that the limiting trait is not the ability to swarm *per se* but rather utilization of the public good. These species could be producing other surfactants that *B. subtilis* cannot use, or the signaling properties of surfactin (34) may be lacking in these species. Comparing this data set to the interaction phenotype results above shows that the species with usable surfactant tended to have antagonistic interactions, especially in the swarm interaction assay, whereas species without a usable surfactant had random scores centered around the varied phenotype (Fig. 5C). These correlations are statistically significant for both the final overall phenotype (*r* = 0.503, *P* = 0.0281) and the swarm assay (*r* = 0.6282, *P* = 0.0040).

To directly test whether antagonistic kin discrimination behavior prevents exploitation of secreted surfactants, we modified the assay by mixing each surfactant-producing strain with the *B. subtilis* mutant on a swarm-inducing medium (Fig. 5D). This approach ensures that the public good under investigation is encountered at the same time as the antimicrobial molecules mediating kin discrimination, which include contact-dependent mechanisms (9). As above, the expression of YFP in *B. subtilis* NCIB 3610 *ΔsrfAA* allowed us to visualize its presence in the swarm and determine the total surface area that it was able to cover.

By itself, the *ΔsrfAA* mutant cannot swarm (area, ≤0.28 cm^2^), but when mixed with its wild-type parent, it is seen throughout the swarm (57 cm^2^, Fig. 5D, second row), indicating effective stealing of the common good. When mixed with other strains, however, *B. subtilis ΔsrfAA* did not spread far in 42 of the 61 combinations (≤1.1 cm^2^, Fig. 5D, third row), likely due to complete killing of either *B. subtilis* (resulting in no YFP signal) or the other strain (resulting in no spread). The mutant exhibited moderate spreading in eight of the mixtures (2.3 to 11 cm^2^, Fig. 5D, fourth row) and robust or complete spreading in 11 mixtures (≥23 cm^2^). Many of the combinations that elicited the best spreading segregated themselves spatially, however, with each strain in distinct sectors or even halves of the plate (Fig. 5D, last row). This segregation did not prevent exploitation of surfactant in this assay but likely prevents stealing of less diffusible molecules like the extracellular matrix components, as the sectors displayed distinctly different morphologies. In summary, antagonistic strains drastically limited the ability of an exploitative mutant to steal surfactant when mixed together (Table 2).

**TABLE 2:** Mixtures of nonkin strains with *B. subtilis* NCIB 3610 *ΔsrfAA*, categorized by the area covered by the mutant

Category	*n*	Area of *ΔsrfAA* spread (cm^2^)			
		Range	Median	Avg	SEM
Zero/little spread	42	0–1.1	0	0.23	0.050
Modest spread	8	2.3–11	6.9	6.4	1.2
Robust spread	11	23–57	28	38	4.5
All nonkin	61	0–57	0.48	7.8	2.0

**FIG 6:**
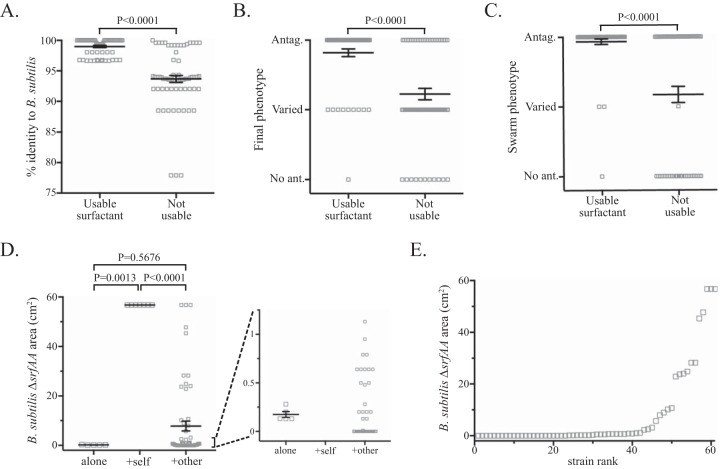
Analyses of surfactant-stealing results. (A to C) Graphs of 16S rRNA gene identity to *B. subtilis* (A), final interaction phenotypes (B), and swarm interaction phenotypes (C) of strains that did or did not have surfactant stolen in the surfactant-stealing assay. The varied phenotypes in the swarm interaction assay are the strains that gave different results on different media, as in Fig. 3B. (D) Total area occupied by *B. subtilis ΔsrfAA* when alone or when mixed with surfactant-producing self (wild-type *B. subtilis* NCIB 3610) or other strains with usable surfactants. The crowded values near the bottom are shown in an expanded scale on the right. (E) Area of *B. subtilis ΔsrfAA* in mixtures with other strains, arranged in increasing order to highlight the large number that showed very little spreading. For all graphs, long horizontal lines are the averages, error bars represent the standard errors of the means, and *P* values from Mann-Whitney tests comparing each set are given above the graphs. “No ant.” and “Antag.” are the no-antagonism and antagonism categories, respectively.

Comparing the strains with and without usable surfactant revealed significant differences in both relatedness and overall interaction phenotypes. The average 16S rRNA gene identity to *B. subtilis* of the surfactant-producing species was 99.00% (97.73% if we exclude the *B. subtilis* strains and focus only on interspecies interactions), whereas “not usable surfactant” species averaged only 93.68% identity to *B. subtilis* (Fig. 6A). Strains that elicited spreading of the mutant were also significantly more likely to have antagonistic interactions (Fig. 6B), and 65% of antagonism strains produced usable surfactant while only 21% of varied and 8% of no-antagonism strains did. Tellingly, 60 of the 61 strains with usable surfactant formed a clear boundary with *B. subtilis* in the swarming assay in Fig. 2, while only 42 of the 71 strains with no usable surfactant formed swarm boundaries (Fig. 6C). In the mixed-swarm assay, the area encompassed by *B. subtilis ΔsrfAA* in mixtures with other strains was significantly different from the combinations with the wild-type parent but not statistically different from the area of the mutant alone (Fig. 6D). The average area covered by the mutant in mixes with other strains was 7.8 cm^2^, but this was heavily influenced by the high-spreading outliers, as the median area is only 0.48 cm^2^ (Table 2). This can be seen either by zooming in on the lower section of the graph (Fig. 6D, right graph) or by ordering the results from low- to high-spreading strains (Fig. 6E). We thus suggest that the species that are still physiologically similar enough to be susceptible to antimicrobial antagonism (nonkin) are also similar enough to exploit certain public goods, but the antagonistic molecules prevent this by killing the other cells before they can benefit from the cooperative molecules (though we have not formally ruled out a shared phylogeny as the cause behind the correlation of antagonism and public good compatibility).

## DISCUSSION

In order to find the phylogenetic range of kin discrimination activity, we tested how a collection of strains from diverse species interacted with a reference strain of *B. subtilis* in multiple multicellular interaction assays. We found that *B. subtilis* maintains antagonism toward virtually all (63/64) of the strains within the *subtilis* clade, indicating that nearby species are strictly designated nonkin. This became less strict in the nearby *pumilus* group and faded completely by the *cereus* clade, where strains displayed the same distribution of interaction types as distant outgroups.

**FIG 7:**
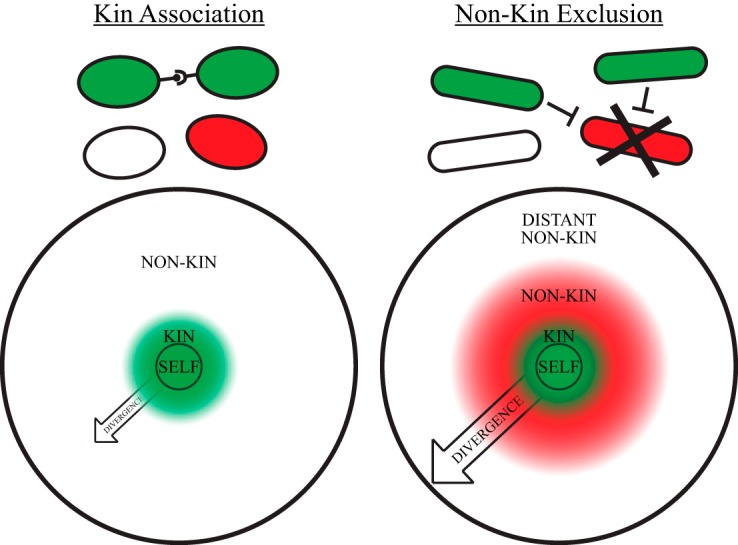
Model of the two types of kin discrimination systems. The depiction of which cells are targeted in each system is at the top. Large circles at the bottom represent phylogenetic distance: highest relatedness in the center, decreasing outward along the radius. Both systems restrict interactions to clonemates (“self”) and kin (green central circle). The kin association system is based on association with kin cells and therefore treats all other species as not kin regardless of their evolutionary distance or physiological similarity (white outer circle). The nonkin exclusion system used by *B. subtilis* is further able to distinguish between closely (red) and distantly (white) related species. Arrows pointing outward indicate selective pressure to diversify, which we hypothesize is stronger in the nonkin exclusion system.

This investigation of where kin discrimination behavior ends could be addressed in *B. subtilis* because it relies on targeting and exclusion of nonkin rather than specific association with kin cells, as most other social microbes do (Fig. 7). In the latter systems, the endpoint of kin discrimination will always be at the kin/nonkin border (likely within the same species), making no further distinctions among nonkin. We found, though, that *B. subtilis* behaves differently toward close versus distant species, an unappreciated consequence of its multicellular lifestyle that likely impacts many aspects of its ecology.

The cutoff point for kin discrimination behavior corresponded to those species that secreted a surfactant molecule that a nonswarming mutant of *B. subtilis* could potentially benefit from (i.e., steal) but was prevented from doing so when in close contact. (Note that we have demonstrated only stealing by *B. subtilis*, not stealing of *B. subtilis*, but we assume that other surfactant-compatible species could reciprocate the exploitation.) There is thus a good correlation between species that can exploit public goods and antagonism between them. This suggests to us that the two could be linked: either the compatibility of public goods impacts antimicrobial range, or the antimicrobial range (selected for other reasons) affects the use of certain public goods. Alternatively, the two traits could arise independently from their shared correlation with phylogeny (and thus general physiology), which may require studies in other bacteria to definitively determine. Likewise, the incompatibility of public goods could merely be a result of evolutionary drift as a consequence of species evolving in isolation from one another and thus under no selective pressure to maintain the same surfactants. More detailed studies of the evolution of kin discrimination genes, public good genes, and the phylogenetic background in which they appear will hopefully shed light on the possible coevolution of these genes separate from their inherent history. It would be particularly interesting to compare the rate of change of the lipopeptide synthetases that produce surfactants to the spectrum of antimicrobials made by each species.

It is also interesting that identical alleles of the quorum-sensing peptide ComX in *Bacillus* can be found in the same phylogenetic range as nonkin designations—*B. subtilis* 168 and *B. mojavensis* RO-C-2 share the same pherotype (for example, see reference 41). ComX controls production of many communal traits, including production of surfactin and extracellular matrix (34), and bacterial quorum sensing is often susceptible to cheating (42–44). Extending kin discrimination to nearby species could thus protect other social behaviors and prevent unintended cross-species communication.

Our results indicate that the very nature of the multicellular lifestyle in *B. subtilis* may be the reason that it uses a nonkin exclusion system instead of kin association. Biofilms and swarms are aggregative types of multicellularity in which any cells in the immediate area that are similar enough physiologically could be incorporated. This is in contrast to organisms like the social ameba *D. discoideum* that form much more precise structures such as fruiting bodies supported by a stalk. *D. discoideum* uses a kin association system to fish out other kin cells and form a mostly clonal fruiting body (12). Cheating in this type of system is mostly limited to signaling mutants that preferentially form spores instead of inviable stalk cells during development (7, 8), a very localized behavior that might not be easily exploited by other species. Biofilms, however, contain many secreted molecules that could provide communal protection to surrounding cells, potentially requiring the more aggressive nonkin exclusion system to safeguard them from exploitation.

Another consequence of the nonkin exclusion system is the ability to coexist in multispecies communities with distant relatives. Instead of living only among kin (in fruiting bodies, for example), the ability to distinguish between close and distant relatives allows *B. subtilis* to form a biofilm surrounded by other species while still guarding against potential exploitation that could undermine the community. Much research has shown that more diverse communities are more productive and more resistant to stresses and pathogens (26), and strain combinations that are less antagonistic are better able to colonize and protect plant roots from pathogen invasion (45).

The final difference between the two kin discrimination systems is their impact on speciation. Nonkin exclusion systems set up a bull’s-eye model (Fig. 7): clonemates and immediate kin (green central circle) are cooperated with until they gain or lose antimicrobial genes and are treated as nonkin (red intermediate circle). Then, over enough time they will diverge further until they are no longer a threat to the cooperative system (white outer circle), at which point the selection pressure to maintain antagonism is relaxed. There is thus a constant push outward to keep the kin group updated by changing both the antimicrobial complement and the cooperative goods utilized. Kin association systems, on the other hand, are a more simplified bull’s-eye: they are under diversifying selection to update the definition of kin, but they do not have a second pressure to set the range of nonkin designation. We expect that this has affected the divergence rates of strains and species in the two systems, though this has not been explicitly examined. We do note, however, that none of the 35 *B. subtilis* strains isolated in this study treated our reference strain NCIB 3610 as kin, indicating that cooperation might be restricted to clones or that only highly sympatric populations contain kin (10).

We expect that nonkin exclusion systems will be more prevalent than kin association systems in bacteria. One reason is that many bacteria are known to aggregate in biofilm-like structures that would benefit from nonkin exclusion, as explained above. The other reason is that this system (in *B. subtilis* at least) arises naturally from possession of narrow-spectrum antimicrobials (9). Many antibiotics are known to be biased toward phylogenetic neighbors (46), which is often explained in terms of niche competition. However, our results suggest that protection of cooperative traits may have an impact on antibiotic spectrum, too, or at least that antibiotic spectrum impacts cooperative trait protection. This is further evidence that we should be looking to nearby species for molecules to specifically target bacterial infections rather than using broad-spectrum antibiotic treatments.

## MATERIALS AND METHODS

### Strains used and soil isolations.

All 191 strains tested for interactions are listed in Data Set S1 in the supplemental material. These included 38 previously isolated strains (mostly from the Bacillus Genetic Stock Center and the American Type Culture Collection) in addition to the 153 isolated for this study. Strains of *B. subtilis* NCIB 3610 deleted for *srfAA* or expressing fluorescent proteins were made in prior studies (10, 11).

New strains were isolated by spore selection from soil samples from five locations: Cambridge, MA (42°22′15.9″N, 71°06′24.3″W); Boston, MA (42°20′26.0″N, 71°06′38.0″W); Asheville, NC (35°29′12.0″N, 82°30′25.5″W); New York, NY (40°46′32.0″N, 73°57′58.0″W); and Adirondack Park, NY (44°06′20.0″N, 73°54′02.0″W). To kill all nonspores, between 0.1 and 0.3 g of soil was suspended in 1 to 3 ml of 0.85% NaCl and heated at 80°C for 15 min. After soil was cooled to room temperature, 200 µg/ml cycloheximide was added to kill all spore-forming eukaryotes. These bacterial spore-enriched mixtures were then spread on a variety of media to obtain a diverse cross section of *Bacilli*: LB, tryptic soy agar (TSA), B medium with 0.7% agar (swarming medium), MSgg (biofilm-promoting medium), and M9mg with 0.7% or 1.5% agar (M9 salts plus 2% mannitol and 0.1% glutamate). Most plates were incubated at 30°C, but a few plates were incubated at room temperature (growth was much slower) or 37°C (growth was much less diverse). Colonies were picked with the goal of maximum diversity, streaked three times on LB at 30°C, and frozen in 20% glycerol. Species were assigned based on the closest match to 16S rRNA gene sequences obtained by performing PCR on isolated genomic DNA using primers 27F (AGAGTTTGATCCTGGCTCAG) and 1492R (TACGGCTACCTTGTTACGACTT). In total, 153 new isolates were used in the interaction assays: 82 from Cambridge soil (LN strains), 58 from Asheville (WNC strains), 7 from Boston (EN strains), 4 from Adirondack (HAY strains), and 2 from New York City (NYC strains).

### Interaction assays.

Interactions between swarms were tested by spotting 2 µl of liquid LB cultures at an optical density at 600 nm (OD_600_) of 0.5 onto 0.7% agar LB or B medium (9). Plates were dried in a laminar flow hood for 30 min and then incubated in a sealed container overnight at 30°C (or sometimes 37°C for the strains in the *subtilis* clade known to grow well at higher temperatures). Strains incapable of spreading across the low-agar surface were allowed to grow alone for 1 day before *B. subtilis* was spotted onto the plate. Swarming proficiency in Table 1 was judged by the ability to spread over 0.7% agar in isolation on either medium type and does not include those strains that spread only in the presence of other swarmers (four strains). Surfactant-stealing assays were done by spotting *B. subtilis ΔsrfAA P_cIo3_-YFP* 3 cm away from the tested strain on 0.7% agar LB or by mixing *B. subtilis ΔsrfAA P_cIo3_-YFP* with each strain in equal concentrations and spotting the mixture in the center of an 0.7% agar LB plate.

Biofilm interactions were done on MSgg medium (29) at 30°C. Strains were initially grown alone to assess their ability to grow on this medium, and slow-growing strains were pregrown up to 3 days before spotting *B. subtilis* 1.5 cm away and growing the strains for an additional 2 to 7 days. Halo assays were performed on the same MSgg medium, except that *B. subtilis* was first top spread by plating 0.5 µl of liquid LB culture (OD_600_ of 0.5, diluted in 100 µl water). After drying, 6 µl (OD_600_ of 0.5) of each tested strain was spotted, and the plate was incubated at 30°C for 14 to 24 h. These amounts of *B. subtilis* and the test strains were found to balance the growth of the lawn and colony to optimize halo formation.

Identification of each strain in all the interaction assays was aided by the constitutively fluorescent *P*_*cIo3*_-*YFP* and *P_hyperspank_-mKate2* constructs. Interactions were imaged with a fluorescent stereoscope and analyzed with ImageJ software (http://imagej.nih.gov/ij).

### Bioinformatic and statistical analyses.

Phylogenetic trees were created in Mega 6.06 using 16S rRNA gene sequences from the type strains of each species (accession numbers and strain names are listed in Data Set S1) since they had longer sequences (at least 1.4 kb) that provided better resolution. Percent identities to *B. subtilis* 16S rRNA genes were obtained from T-Coffee multiple sequence alignments (http://www.ebi.ac.uk/Tools/msa/). In Fig. S2, BLASTp was used to search for homologs using the following protein sequences as bait: SrfAA from *B. subtilis* NCIB 3610, LicB from *B. licheniformis* ATCC 10716, BAT_3766 from *B. pumilus* ATCC 7061, ItuA from *Bacillus subtilis* subsp. *inaquosorum* KCTC 13429, FenD from *B. subtilis* F-29-3, EpsD from *B. subtilis* NCIB 3610, and TasA from *B. subtilis* NCIB 3610. A hit was considered a true lipopeptide synthetase homolog if it covered >90% of the protein and had >70% amino acid identity, a partial homolog if it had 50 to 70% identity, and a nonhomolog if it had <50% identity or <90% coverage; true homologs of matrix proteins covered >90% of the protein and had >60% amino acid identity, a partial homolog had 30 to 60% identity, and nonhomologs had <30% identity or <90% coverage. These cutoffs were based on searches for the nearest paralog with different function (i.e., a different lipopeptide synthetase).

All statistical analyses were performed in GraphPad Prism 6.0 after converting interaction phenotypes into numerical values (antagonism, 1; varied, 0.5; no antagonism, 0). All Mann-Whitney tests were two-tailed and assumed nonparametric distributions of data sets.
